# Effect of the synthesis of rice non-symbiotic hemoglobins 1 and 2 in the recombinant *Escherichia* *coli* TB1 growth

**DOI:** 10.12688/f1000research.7195.2

**Published:** 2016-03-01

**Authors:** Emma Álvarez-Salgado, Raúl Arredondo-Peter

**Affiliations:** 1 Laboratorio de Biofísica y Biología Molecular, Centro de Investigación en Dinámica Celular, Instituto de Investigación en Ciencias Básicas y Aplicadas, Universidad Autónoma del Estado de Morelos, Cuernavaca, Morelos, 62210, Mexico

**Keywords:** Function, heterologous expression, in vivo, Oryza, oxygen

## Abstract

Non-symbiotic hemoglobins (nsHbs) are widely distributed in land plants, including rice. These proteins are classified into type 1 (nsHbs-1) and type 2. The O _2_-affinity of nsHbs-1 is very high mostly because of an extremely low O _2_-dissociation rate constant resulting in that nsHbs-1 apparently do not release O _2_ after oxygenation. Thus, it is possible that the *in* *vivo* function of nsHbs-1 is other than O _2_-transport. Based on the properties of multiple Hbs it was proposed that nsHbs-1 could play diverse roles in rice organs, however the *in* *vivo* activity of rice nsHbs-1 has been poorly analyzed. An *in* *vivo* analysis for rice nsHbs-1 is essential to elucidate the biological function(s) of these proteins. Rice Hb1 and Hb2 are nsHbs-1 that have been generated in recombinant *E*s *cherichia* *coli* TB1. The rice Hb1 and Hb2 amino acid sequence, tertiary structure and rate and equilibrium constants for the reaction of O _2_ are highly similar. Thus, it is possible that rice Hb1 and Hb2 function similarly *in* *vivo*. As an initial approach to test this hypothesis we analyzed the effect of the synthesis of rice Hb1 and Hb2 in the recombinant *E*. *coli* TB1 growth. Effect of the synthesis of the O _2_-carrying soybean leghemoglobin *a*, cowpea leghemoglobin II and *Vitreoscilla* Hb in the recombinant *E*. *coli* TB1 growth was also analyzed as an O _2_-carrier control. Our results showed that synthesis of rice Hb1, rice Hb2, soybean Lb *a*, cowpea LbII and *Vitreoscilla* Hb inhibits the recombinant *E*. *coli* TB1 growth and that growth inhibition was stronger when recombinant *E*. *coli* TB1 synthesized rice Hb2 than when synthesized rice Hb1. These results suggested that rice Hb1 and Hb2 could function differently *in vivo*.

## Introduction

Non-symbiotic hemoglobins (nsHbs) are O _2_-binding proteins widely distributed in land plants, including rice ^1^. The nsHbs are classified into type 1 and type 2 (nsHbs-1 and nsHbs-2, respectively) based on sequence similarity and O _2_-affinity ^2, 3^. The O _2_-affinity of nsHbs-1 is very high mostly because of an extremely low O _2_-dissociation ( *k*_off_) rate constant ^3– 5^ resulting in that nsHbs-1 apparently do not release O _2_ after oxygenation ^6, 7^. In contrast, the O _2_-affinity of nsHbs-2 is moderate mostly because of a moderate to high *k*_off_ rate constant for O _2_, thus apparently nsHbs-2 easily release O _2_ after oxygenation ^2, 3, 6, 7^. Hence, it is possible that the *in vivo* function of nsHbs-1 is other than O _2_-transport and that nsHbs-2 function *in vivo* as O _2_-carriers.

 Five copies ( *hb1* to *5*) of the *nshb* gene have been detected in the rice genome, which are differentially expressed in embryonic and vegetative organs from plants growing under normal and stress conditions ^8– 11^. Based on the available information on the properties of rice nsHbs and data from the analysis of other plant and non-plant Hbs, it was proposed that rice nsHbs could exhibit a variety of functions *in vivo*, including O _2_-transport, O _2_-sensing, NO-scavenging and redox-signaling ^6, 12, 13^. However, the *in vivo* activity of rice nsHbs has been poorly analyzed ^12^. An *in vivo* analysis for rice nsHbs is essential to elucidate the biological function(s) of these proteins. An approach to analyze the *in vivo* activity of nsHbs is generating knock out rice for individual *nshb* genes, however this is complicated because of the existence of five copies of *nshb* in the rice genome. An alternative approach to analyze the *in vivo* activity of rice nsHbs is examining individual rice nsHbs in a heterologous system, such as recombinant *Escherichia coli*. Rice Hb1 ^4^ and Hb2 ^14^ are nsHbs-1 that have been generated in recombinant *E. coli* TB1. The rice Hb1 and Hb2 amino acid sequence ^4^, tertiary structure ^15^ and rate and equilibrium constants for the reaction of O _2_^4, 14^ are highly similar. Thus, it is possible that rice Hb1 and Hb2 function similarly *in vivo*. As an initial approach to test this hypothesis we analyzed the effect of the synthesis of rice Hb1 and Hb2 in the recombinant *E. coli* TB1 growth. Our results showed that synthesis of rice Hb1 and Hb2 inhibited the recombinant *E. coli* TB1 growth and that growth inhibition was stronger when recombinant *E. coli* TB1 synthesized rice Hb2 than when synthesized rice Hb1.

## Methods

Untransformed (wild-type) and transformed (recombinant) *E. coli* TB1 (Invitrogen, CA, USA) containing the constitutive pEMBL18 ^+^::Hb1 ^4^, pEMBL18 ^+^::Hb2 ^14^, pEMBL18 ^+^::Lb *a* ^16^, pEMBL18 ^+^::LbII ^17^ and pUC18::VHb ^18^ plasmids were grown in LB broth (Sigma-Aldrich, MO, USA) at 37°C with shaking at 200 rpm. Plasmids pEMBL18 ^+^::Lb *a*, pEMBL18 ^+^::LbII and pUC18::VHb were included as an O _2_-carrier control since they code for the synthesis of the O _2_-carrying soybean leghemoglobin *a* (Lb *a*), cowpea leghemoglobin II (LbII) ^17, 19, 20^ and *Vitreoscilla* Hb (VHb) ^21, 22^, respectively. The existence of the VHb insert into the pUC18::VHb plasmid was verified by PCR (30 cycles at 55°C/30s for annealing, 72°C/30s for extension and 95°C/30s for denaturation) using specific oligonucleotides (VitHb/ATG: 5´-ATG TTA GAC CAG CAA ACC ATT-3´ and VitHb/TAA: 5´-TTA TTC AAC CGC TTG AGC GTA-3´) designed from the *vhb* sequence deposited in the Genbank database under the accession number X13516. The existence of the Hb1, Hb2, Lb *a* and LbII inserts into the pEMBL18 ^+^::Hb1, pEMBL18 ^+^::Hb2, pEMBL18 ^+^::Lb *a* and pEMBL18 ^+^::LbII plasmids, respectively, was verified by *Eco*RI- and *Nco*I (Invitrogen, CA, USA) -double digestion. Inserts were detected by electrophoresis in a 1.4% agarose gel. The existence of recombinant Hbs in cell soluble extracts was verified by SDS-PAGE in a 12.5% polyacrylamide gel. Evaluation of the effect of the Hb synthesis in the recombinant *E. coli* TB1 growth was performed in 50 ml cultures inoculated with ≈5 × 10 ^8^ colony forming units from a 20 ml overnight culture. Wild-type *E. coli* TB1 was included as control. All assays were performed in triplicate. Cell growth was quantitated by spectrophotometry using *λ* = 650 nm for an 8.5 h period.

## Results and discussion

**Figure 1.**
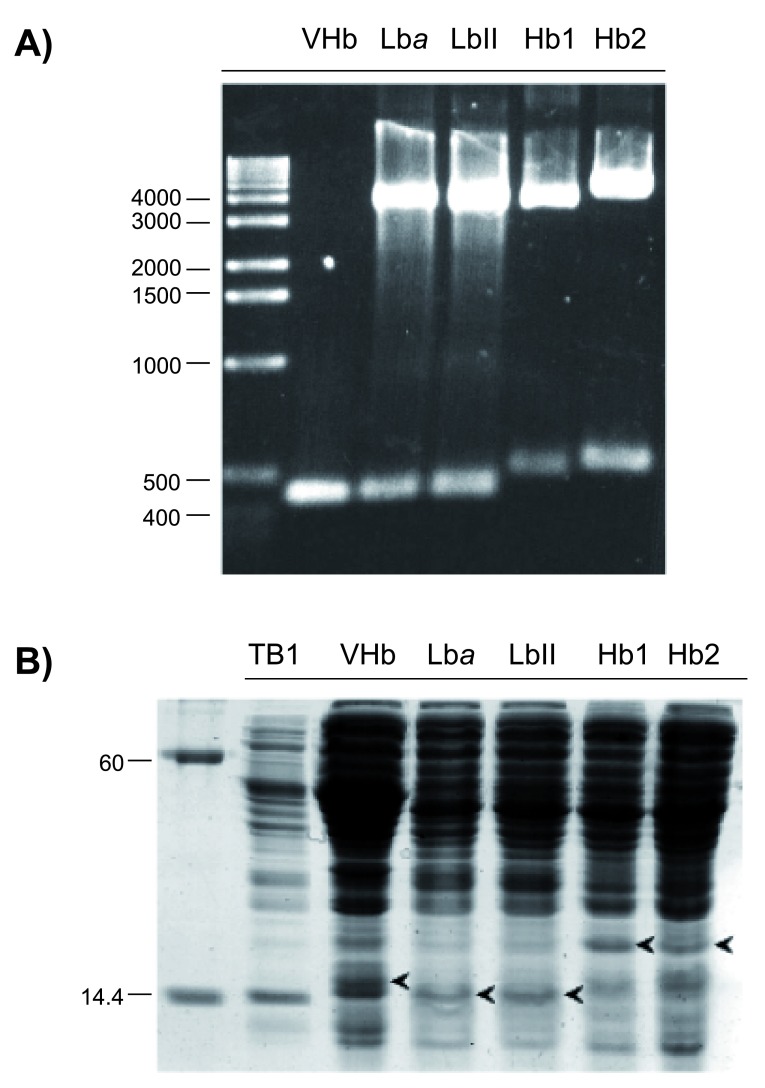
**(A) Detection of *Vitreoscilla* Hb PCR fragment and soybean Lb *a*, cowpea LbII, rice Hb1 and rice Hb2 cDNAs from recombinant *E. coli* TB1 by agarose gel electrophoresis.** PCR fragment and cDNA sizes are within the 435 to 507 base pairs range, which corresponds to the molecular sizes of the Hb cDNAs analyzed here. Molecular size markers are indicated in base pairs. **(B) Detection of *Vitreoscilla* Hb, soybean Lb *a*, cowpea LbII, rice Hb1 and rice Hb2 proteins (arrow heads) from recombinant *E. coli* TB1 soluble extracts by SDS-PAGE.** A 20 to 50 μg aliquot of total soluble proteins was loaded onto each lane. Recombinant Hb masses are within the 14 to 18.4 KD range, which corresponds to the molecular masses of the Hbs analyzed here. Mass markers are indicated in kD.

Electrophoretic analysis of the PCR reaction and *Eco*RI- and *Nco*I-double digestions showed that plasmids isolated from recombinant *E. coli* TB1 contained inserts corresponding to the rice Hb1 ^4^, rice Hb2 ^4^, soybean Lb *a* ^16^, cowpea LbII ^17^ and *Vitreoscilla* Hb ^18^ cDNAs ( Figure 1A). Likewise, analysis by SDS-PAGE showed that rice Hb1, rice Hb2, soybean Lb *a*, cowpea LbII and *Vitreoscilla* Hb existed in the soluble extracts of recombinant *E. coli* TB1 ( Figure 1B). This evidence indicated that rice Hb1, rice Hb2, soybean Lb *a*, cowpea LbII and *Vitreoscilla* Hb were synthesized by recombinant *E. coli* TB1.

**Figure 2.**
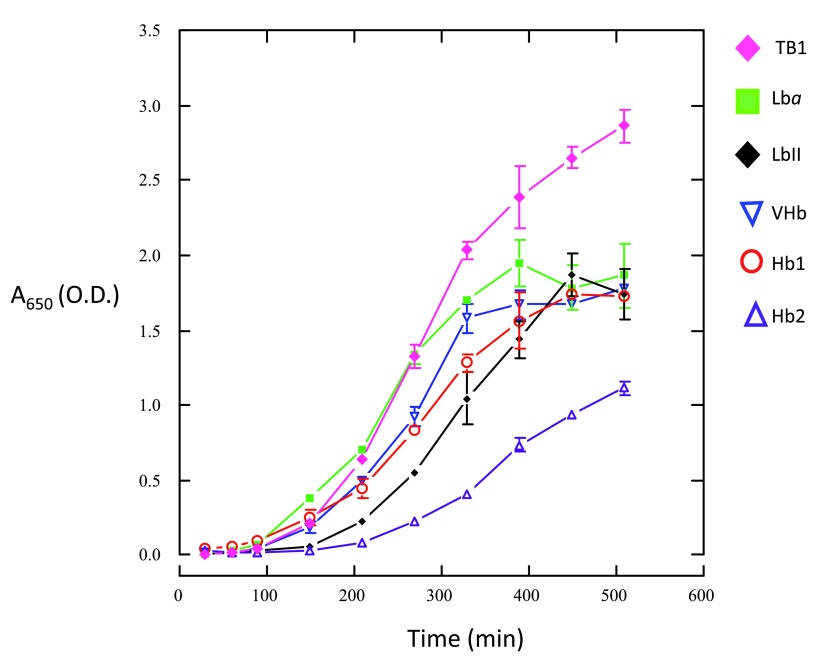
Growth of wild-type (TB1) and recombinant (VHb, Lb *a*, LbII, Hb1 and Hb2) *E. coli*. Values (mean ± SD) correspond to three replicates. See the Methods section for experimental details.

Figure 2 shows that synthesis of rice Hb1, rice Hb2, soybean Lb *a*, cowpea LbII and *Vitreoscilla* Hb inhibited the recombinant *E. coli* TB1 growth. This was unexpected for soybean Lb *a*, cowpea LbII and *Vitreoscilla* Hb because these proteins would promote cell growth due to their O _2_-transport activity ^17, 19– 22^. However, under the conditions tested in this work apparently soybean Lb *a*, cowpea LbII and *Vitreoscilla* Hb affected some aspects of the recombinant *E. coli* TB1 metabolism, possibly owed to the constitutive expression of these proteins into the host cells. Synthesis of rice Hb1 inhibited the recombinant *E. coli* TB1 growth similarly (∼37%) to the synthesis of soybean Lb *a*, cowpea LbII and *Vitreoscilla* Hb. This observation suggests that rice Hb1 could function *in vivo* similarly to O _2_-carrying Hbs. Likewise, synthesis of rice Hb2 also inhibited the recombinant *E. coli* TB1 growth. However, growth inhibition was stronger (∼61%) when recombinant *E. coli* TB1 synthesized rice Hb2 than when synthesized rice Hb1. This observation suggests that rice Hb2 could function *in vivo* by scavenging O _2_, possibly owing to its extremely low *k*_off_ rate constant for O _2_^14^.

## Conclusions

Results presented in this work suggest that in spite of the high similarity between rice Hb1 and Hb2 these proteins could function differently *in vivo*. In order to elucidate the apparent metabolic effects generated by the synthesis of rice Hb1 and Hb2, future work might focus on the physiological and biochemical characterization of recombinant *E. coli* TB1. This may include measuring cell respiratory rates and identifying cell proteins and metabolites using oximetry and proteomic and metabolomic approaches, respectively. Results from these analyses could provide valuable information to understand the *in vivo* function of rice nsHbs.
