# Supplementary material for: Effect of the synthesis of rice non-symbiotic hemoglobins 1 and 2 in the recombinant Escherichia coli TB1 growth
Source: F1000Res. 2016 Mar 1;4:1053. Originally published 2015 Oct 12. [Version 2] doi: 10.12688/f1000research.7195.2 (PMC4776736; doi:10.12688/f1000research.7195.2)
Supplement: Peer review history

# Peer review: Referee response for version 2

**DOI:** 10.5256/f1000research.8834.r12702

**Published:** 2016-03-02

**Authors:** Angel Matilla

**Affiliations:** 1 Department of Plant Physiology, Faculty of Pharmacy, University of Santiago de Compostela, Santiago de Compostela, Spain

The authors have sufficiently addressed my concerns.

I have read this submission. I believe that I have an appropriate level of expertise to confirm that it is of an acceptable scientific standard.

# Peer review: Referee response for version 1

**DOI:** 10.5256/f1000research.7751.r12624

**Published:** 2016-02-23

**Authors:** Angel Matilla

**Affiliations:** 1 Department of Plant Physiology, Faculty of Pharmacy, University of Santiago de Compostela, Santiago de Compostela, Spain

The work by Álvarez-Salgado and Arredondo-Peter (2015) was carefully evaluated. Broadly, this research is worthy of achieving an approval in F1000Research. However, before it achieves this status, is necessary to carry out some minor modifications.

Thus, the first paragraph of the Introduction, referred to the different affinity of nsHbs1 and nsHb2 for O _2_, needs to include some recent references (eg. Hoy and Hargrove, 2008; Smagghe et al, 2009; Thiel et al., 2011; among others). Likewise, in the second paragraph of the Introduction (ie. Based on the available information…. NO scavenging and redox-signaling) the following latest references must also be added (ie. Siddiqui et al., 2010; Vigeolas et al., 2010, among others).

With respect to Res & Discuss, (i) the first paragraph should include some reference to show that the bands referred by the authors (Fig. 1A) specifically belong to rice (Hb1, Hb2), soybean (LBA), cowpea (LbII) and Vitreoscilla (Hb) cDNAs. This fact is key in this work. Likewise, bands corresponding to VHb and Lba (Fig. 1B) are confusing to the reader; (ii) I would eliminate from Fig. 2 the results of LBA, LbII and HBv growth (include as data not shown) because the main importance of this work are the results concerning nb1 and nbII; (iii) "... these proteins would promote cell growth due to their O _2_-transport activity"; this conclusion is based in old results and is very risky; this growth promotion should also be referred to higher plants?; please discuss; and (iv) I repeat, some actual references must be also included into discussion.

I have read this submission. I believe that I have an appropriate level of expertise to confirm that it is of an acceptable scientific standard, however I have significant reservations, as outlined above.

# Peer review: Referee response for version 1

**DOI:** 10.5256/f1000research.7751.r11632

**Published:** 2016-01-06

**Authors:** Robert Hill

**Affiliations:** 1 Department of Plant Science, University of Manitoba, Winnipeg, MB, Canada

This is an interesting approach to possibly differentiating between the functions of this group of proteins.  A fundamental question that arises out of this work is why do hemoglobin proteins inhibit *E. coli *growth? The growth differences between the rice Pgb1.1-transformed line and the rice Pgb1.2 line is particularly interesting. I would be cautious, however, in attempting to interpret the results with respect to the proteins possibly behaving as oxygen carriers/transporters. My concerns are based on the following:

1. Why would a unicellular organism without mitochondria require an oxygen carrier since oxidative phosphorylation occurs on the plasma membrane?
2. If plant phytoglobins have an oxygen carrier function would you not expect the two class 1 phytoglobins to have the same effect since they both have similar oxygen binding characteristics?
3. Why would you anticipate that Pgb1.2 might participate more in NO scavenging than Pgb1.1 in *E. coli* if they both have similar configurations in the heme pocket? Is it not the class 2 Pgbs that are suggested to possibly being less amenable to NO scavenging?
4. Is it possible that the expression of the class 2 protein is interfering with some function of the native flavohemoglobin in E. coli?
5. Although E. coli does not have a true nucleus, is it possible that the protein is specifically interfering with transcriptional/translation functions in the chromosome, e.g., the N-end rule pathway as one possibility?

My other comment concerns the terminology. The individuals who work in this area agreed to forego the use of the term "nonsymbiotic hemoglobins" at an international meeting in 2014, replacing it with "phytoglobin", since the original designation does not appropriately describe the protein. I would hope that the authors consider modifying the manuscript to ensure that the name change becomes recognized in the literature.

I have read this submission. I believe that I have an appropriate level of expertise to confirm that it is of an acceptable scientific standard.

# Response

**Published:** 2016-01-06

**Authors:** Raul Arredondo-Peter

**Affiliations:** Universidad Autonoma del Estado de Morelos, Mexico

We thank Dr. Robert Hill for evaluating this article and providing constructive comments and suggestions.

We agree with Dr. Hill´s comments corresponding to questions 1 to 5. Undoubtedly they should also be considered in future research focused to elucidate the physiological effects of the synthesis of rice non-symbiotic hemoglobins 1 and 2 in recombinant *E*. *coli* TB1.

Regarding the terminology, we decided to not change the term “non-symbiotic hemoglobins 1 and 2” by “phytoglobins 1.1 and 1.2” (which was accepted in the 2014 XVIII Oxygen-Binding and Sensing Proteins meeting) because details for the accepted nomenclature have not been published. Thus, the accepted nomenclature is not yet widely available to individuals working/interested in the plant hemoglobins field. Hence, replacing the term non-symbiotic hemoglobins 1 and 2 (which has been used for many years in the literature) by the novel term phytoglobins 1.1 and 1.2 could result as confusing to readers of this article.
